# Servant Leadership Behavior at Workplace and Knowledge Hoarding: A Moderation Mediation Examination

**DOI:** 10.3389/fpsyg.2022.888761

**Published:** 2022-05-04

**Authors:** Shagufta Zada, Jawad Khan, Imran Saeed, Zhang Yong Jun, Alejandro Vega-Muñoz, Nicolás Contreras-Barraza

**Affiliations:** ^1^Business School, Henan University, Kaifeng, China; ^2^Department of Business Administration, ILMA University, Karachi, Pakistan; ^3^Department of Business Administration, Iqra National University, Peshawar, Pakistan; ^4^Institute of Business and Management Sciences, The University of Agriculture, Peshawar, Pakistan; ^5^Public Policy Observatory, Universidad Autónoma de Chile, Santiago, Chile; ^6^Facultad de Economía y Negocios, Universidad Andres Bello, Santiago, Chile

**Keywords:** mastery climate, psychological safety, knowledge hoarding, servant leadership, workplace

## Abstract

Servant leadership practice honesty, stewardship, and high moral standards while prioritizing the needs of subordinates. The moral concern of a servant leadership is to support others and put the needs of others first. We investigated the relationship between servant leadership, psychological safety, and knowledge hoarding in accordance with social learning theory in a survey of 347 workers across 56 teams. The results of this study illustrate that servant leadership is negatively associated with knowledge hoarding and positively associated with psychological safety. We also found that a mastery climate moderated the relationship between servant leadership and knowledge hoarding. This study highlights the theoretical and practical implications that contribute to the body of knowledge. It helps organizations that the presence of servant leadership may discourage knowledge hoarding by providing a psychologically safe mastery climate.

## Introduction

Employees who hide, hoard, or simply refuse to share knowledge with others in their organization are becoming a rising issue in today’s workplace. It is disruptive and has a significant impact on the lack of productivity in the workplace (Flynn et al., 2022). It seems that employees who purposefully hoard knowledge will be met by similar selfish conduct on the part of their coworkers, which will eventually harm them and reduce their ability to be creative (Wu J. et al., 2021). Organizations are developing new working methods. Our typical business problems are layered with additional challenges: new ways of functioning, keeping employee’s safe and addressing layoffs, furloughs, and loss of revenue (Newman and Newman, 2021). Negative consequences on the global economy have adverse social implications (i.e., good health and well-being, poverty, quality education, etc.). We need servant leadership that helps employees emotionally and cognitively to survive and face all those challenges efficiently (Obi et al., 2020). Servant leader’s primary moral objective and obligation are to serve their employees (Lumpkin and Achen, 2018). They put the needs of their subordinates first, rather than their own self-interests (Hunter et al., 2013). Leaders who practice servant leadership make certain their subordinates in developing their career professionally and even in terms of their physical well-being (Latif and Marimon, 2019). Leaders who transfer their services to their workers are more likely to develop talented, knowledgeable, and motivated individuals who, in turn enhance the overall operations and management of the organization (Abdulmuhsin et al., 2021). Scholars have studied servant leadership and its positive effect on employees and organizations extensively in the past (Saleem et al., 2020). Servant leadership was positively related to employees work engagement, workplace spirituality, work motivation, individual and team performance, and organization effectiveness (Baloch et al., 2021). Servant leadership also plays a crucial role in reducing employee’s turnover, CWB, employee cynicism, and job stress (Erkutlu and Chafra, 2017). Previous studies on servant leadership and knowledge management have been divided (Hunter et al., 2013; He et al., 2021). Most studies have examined the relationship between servant leadership and employee knowledge sharing behavior, but there is a dire need of to examine servant leadership with knowledge hoarding behavior. Knowledge hiding and knowledge hoarding are two different concepts, knowledge hiding is intentional act to hide and conceal knowledge when someone request while knowledge hoarding is purposely keeping information and knowledge to themselves.

According to a poll of 1700 newspaper readers conducted by The Globe and Mail, employees are more prone to hoard knowledge from their coworkers than sharing it publicly. A similar study conducted in China, 46% of those polled admitted that they hoarding knowledge at their work place (Peng, 2013). For Fortune 500 businesses, this turn in to a yearly loss of $31.5 billion in revenue Babcock (2004). Organizations face a huge cost of knowledge hoarding; therefore, leaders must figure out to prevent it from happening in their organizations. When describing unethical conduct in organizations, in such situation servant leadership is one of the good choice (Wah et al., 2007; Lumpkin and Achen, 2018). Servant leaders may positively influence their teams’ moral standards by serving as positive role models, enforcing better moral standards *via* the use of punishments and incentives, and showing concern and care for their workers (Hunter et al., 2013; Sun et al., 2020b). In general, most employees consider it unethical and detrimental to the interests of the company and its employees to hoard knowledge (Serenko, 2019). Knowledge hoarding may also be deemed improper in an atmosphere characterized by high service levels (Oliveira et al., 2021).

This research was based on Bandura’s social learning theory (Bandura, 1977) and evaluated a connection between servant leadership and knowledge hoarding in the workplace. According to social learning theory, individuals try to follow leader’s behavior and actions in the workplace (Wu J. et al., 2021). Servant leadership communication with their subordinates regarding what is wrong or right through open communication (Latif and Marimon, 2019; He et al., 2020). Therefore, social learning theory is helpful to explain the social learning process through which followers adopt the learning approach (Wu J. et al., 2021). This approach helps employees to less hoard their knowledge under servant leadership. Attitude, subjective norms, and perceived behavioral control all play a key role in employees’ desire to share their expertise with their co-workers in a servant leadership style. Understanding how servant leadership impacts workers’ knowledge-hoarding behavior is based on findings from social learning theory (Bandura, 1977). It also looks at how servant leadership affects employee knowledge hoarding *via* psychological mechanisms. It is more probable that employees will have a high level of psychological safety when their supervisors exhibit servant leadership by emphasizing mutual respect which is beyond the interpersonal trust.

Psychological safety—“Psychological safety is a multi-dimensional, dynamic phenomenon that concerns team members’ perception of whether it is safe to take interpersonal risks at work” (Liang et al., 2012; Ali et al., 2021). Additionally, by emphasizing psychological safety as a critical motivator for workers to express themselves, share their ideas, and exchange knowledge (Iqbal et al., 2020). Here in this study, we examine the servant leadership role in psychological safety, which we further studied with knowledge hoarding. A necessary boundary condition of the supposed causal chain is also identified, further developing our servant leadership model and knowledge hoarding. In terms of knowing how to prevent knowledge hoarding occurring from the organization’s perspective, creating a mastery climate is essential. Knowledge hoarding is done for three reasons: (1) employees hoard knowledge so that they become irreplaceable. (2) It might be nerve-wracking to put oneself out there. What if your coworkers or superiors have anything terrible to say about you? (3) Employees will be less inclined to divulge their “secrets” if your company favors individual achievements over collective ones. Social learning and psychological well-being are essential (Sendjaya et al., 2019; Saeed et al., 2022a). A mastery climate, in which workers’ efforts, collaboration, understanding, and self-development are valued, is also assumed by theorists while developing their ideas.

Employees may see knowledge hoarding as a detrimental behavior in a mastery climate since it hinders the reciprocal advantages of knowledge sharing, such as developing skills in their teams (Bari et al., 2019). The research on knowledge hoarding has also emphasized the importance of mastery climate as a critical contextual moderator (Caniëls et al., 2019; He et al., 2019). As a result, we propose exploring the moderating function of mastery climate to determine the boundary conditions of the servant leadership–knowledge concealment relationship. Furthermore, our theoretical viewpoint and empirical findings make significant contributions to the literature on organizational behavior and knowledge management, both of which are key areas of study in their respective fields. The relation between servant leadership and knowledge hoarding is limited and has not been studied in the past. Therefore, studying the role of servant leadership with knowledge hoarding is our main of the research, and linking the mechanism between servant leadership and knowledge hoarding is limited. Abdullah et al. (2019) examined the direct link between ethical leadership and knowledge hoarding in the past. Still, this study is novel to explore the servant leadership role and mediation (psychological safety) and moderating role of (i.e., mastery climate). The target population was students in laboratory settings in previous studies, but this study used full-time employees from actual work settings.

**FIGURE 1:**
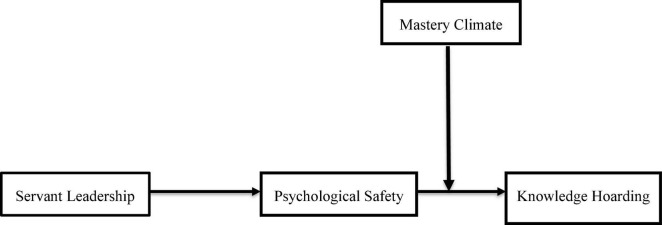
Conceptual model.

Researchers believe that activities carried out in laboratories may fail to elicit the kinds of solid affective reactions needed to uncover the underlying causes of immoral behavior since they are not stimulating enough (Shin, 2014). A further limitation of laboratory testing may be that it cannot accurately recreate the long-term connections and dynamics that occur in real-world work scenarios (Tsai et al., 2012). Thus, in this research, we are interested in determining how and when servant leadership is associated with confidential information in the workplace. First, our data show that servant leadership and information hoarding negatively correlate. Second, a mediation framework is developed based on social learning theory that connects servant leadership to knowledge hoarding *via* psychological safety. Third, mastery climate was examined as a boundary condition between negative association of servant leadership and knowledge hoarding. Fourth, to affect knowledge hoarding, we evaluate the connection between psychological safety and mastery atmosphere. Additionally, the cross-level design and the two-phase data gathering technique were used in this work, which both contribute to the production of more relevant and dependable results. Our study model is shown in Figure 1.

## Theoretical Background and Hypotheses

### Servant Leadership and Knowledge Hoarding

Servant leadership is defined as “the demonstration of normatively appropriate conduct through personal actions and interpersonal relationships, and promoting such conduct to followers through two-way communication, reinforcement, and decision-making” (Reed et al., 2011). Andersen (2018) defined that servant leadership consists of two essential attributes. The first one is related to their moral conduct, where servant leaders have trustworthiness, caring, and stewardship qualities. Second the management component, wherein service leaders influence their followers through their actions, encourage helping behavior in the organization, and discourage immoral behavior (Andersen, 2018). Establishing high standards for followers and mentoring them to keep them accountable for acceptable behavior while still treating them equitably (Latif and Marimon, 2019). According to social learning theory, social conduct is learnt through observing and copying the behavior of others in social situations. The technique of mentorship, which is explained by social learning theory, allows servant leaders to intentionally or unintentionally affect the conduct of their subordinates in this manner (Bandura, 1977). Social learning theory helps us to analyze the relationship between servant leadership and knowledge hoarding. Following social learning theory, people gain knowledge through the actions and behaviors of their mentorship. Through this role-modeling process, individuals learn appropriate behavior and activities that help them act decently. According to Liden et al. (2014), leaders’ show and serves moral conduct and influence others through punishment and rewards approaches. Such an approach is reliable in followers in inducing moral behavior. They are seen as appealing and credible role models because of their position as servant leaders in organizations. When it comes to employees, servant leaders provide employees significant hints about how they might act in a servant-like manner instead of engaging in unethical practices like knowledge hoarding (Song et al., 2015). Employees that follow a servant leader are more likely to engage in the servant or good behaviors (Iqbal et al., 2020). Because of this, servant leaders may give incentives to their employees for participating in cooperative behaviors such as knowledge sharing while discouraging immoral behaviors such as knowledge hoarding (Iqbal et al., 2020). In sum, it is stated that there is a negative relationship between servant leadership and knowledge hoarding by influencing or adequately helping followers. Through their actions, followers can differentiate between wrong and right in the workplace. To be a servant leader, one must put the interests of others instead of one’s own, demonstrate empathy and care, and work for the betterment of their team members and organization (Wu J. et al., 2021). As they develop connections with their subordinates and provide developmental opportunities, servant leaders may help their organizations successfully implement knowledge management practices (Abdulmuhsin et al., 2021).

**Hypothesis 1:** There is a negative association between servant leadership and knowledge hoarding.

### Servant Leadership and Psychological Safety

Workers’ psychological safety will improve in the presence of servant leadership. Leaders who have servants behavior follow the workplace rules and treat others how they want to be treated (Erkutlu and Chafra, 2017). When leaders practice servant leadership, they exhibit sensitivity, thoughtfulness, and caring for their employees by reminding them that their first duty is the psychological safety of their subordinates (Andersen, 2018). It is believed that servant leaders’ actions may “trickle-down” to their subordinates. According to social learning theory, examines how both environmental and cognitive variables interact to impact human learning and behavior in order to understand how people learn and behave, which in turn may encourage others who observe the generally uniform acts of servant leaders toward their colleagues to follow their example (Martin et al., 2016). When servant leaders engage honestly and openly with their workers, they create a win-win scenario for everyone involved. Mutual respect and inter-personal trust emerge between leaders and their followers due to this connection (Obi et al., 2020). Additionally, the past study has shown that when employees see servant leaders’ interpersonal behaviors like compassion, excitement, devotion, and empathy, they feel more psychologically safe (Ma et al., 2021). Employees’ psychological safety is increased by servant leaders, who create an environment where they feel comfortable expressing their thoughts views and making choices (Ma et al., 2021; Saeed et al., 2022a,b). According to Edmondson (1999), people in a condition of psychological safety are characterized by their ability to be engaged, respected, and cherished. They are confident that if they speak out, ask questions, or make mistakes, they will not be embarrassed, sidelined, or penalized in any way. It is a safe space where servant leaders may be open and honest with their followers. In empirical study shows that psychological safety is associated with servant leadership (Brohi et al., 2021; Khan et al., 2022b), and it shows that it is an essential psychological mechanism in organizations (Brohi et al., 2021).

**Hypothesis 2:** There is a positive association between servant leadership and psychological safety.

### Psychological Safety and Knowledge Hoarding

Knowledge hoarding—“when employees purposely keep critical knowledge to themselves—is a fairly common phenomenon found in companies of all sizes.” It’s an uphill battle to create a culture of knowledge sharing (Connelly et al., 2012). It is common for employees to keep their knowledge hidden from one another, and the level of trust between coworkers influences how each replies when asked for knowledge in the workplace (Connelly et al., 2012). There are two reasons why knowledge hoarding is negatively associated with psychological safety. First, psychological safety is a consequence of mutual respect and trust between people who are close to one another, which is a key aspect that is the opposite of hoarding knowledge (Connelly et al., 2012). Psychological safety refers to the degree to which a person feels free to be open and honest about their feelings and actions without fear of repercussions to their self-perception, social standing, or professional prospects. People are more likely to feel psychologically secure when they have connections with their coworkers based on mutual trust and support (Kahn, 1990). Having excellent psychological safety means that individuals may trust their colleagues and not be ashamed or penalized for expressing themselves since interpersonal situations are not harmful (Zhang and Bartol, 2010). Instead, someone who has a low sense of psychological safety may develop sentiments of distrust toward their coworkers, believing that they would do them harm (Connelly et al., 2012). Obi et al. (2020), have claimed that interpersonal mistrust can affect an individual’s knowledge hoarding practices. The inability to place faith in one’s coworkers may lead to hoarding information from one’s colleagues, which indicates poor psychological safety. Second, high levels of psychological safety encourage employees to share work-related knowledge with others and are less afraid of the recipient’s feedback (Zhao and Jiang, 2021; Zada et al., 2022a). Ehrhart (2004) argues that having regular conversations with coworkers on work-related events promotes the development of shared meanings and collective assessments of workplaces. Thus, the likelihood of employees expressing opinions with one another and fostering a culture of knowledge sharing amongst themselves increases when they feel comfortable and safe in their workplace (Connelly et al., 2012).

**Hypothesis 3:** There is a negative relationship between psychological safety and knowledge hoarding.

### The Mediating Role of Psychological Safety

The emotional trust between leaders and followers must be considered when evaluating the quality of social interaction between the two parties. For leaders to be trusted by their followers, trust creates the strong bond between leader and their followers. Trust in the leader has a favorable impact on various outcomes, including organizational citizenship behavior, performance, and satisfaction (Chughtai, 2016; Sun et al., 2020a). According to Edmondson and Lei (2014), one of the most critical factors that contribute to psychological safety is a workplace that encourages open communication and mutual respect amongst co-workers and the ability to share information (Ullah et al., 2021; Khan et al., 2022a). Many studies have also stated that leaders who demonstrate an embodied service attitude and create an atmosphere of service help their employees to experience psychological safety (Liden et al., 2014; Zada et al., 2022a,b). Having a psychologically safe environment would alleviate any concerns about team members’ reactions that make the member feel ashamed or frightened. In an environment where people feel safe and do not fear the ramifications of taking interpersonal risks, people are less inclined to hoard knowledge. A servant leadership create good environment which fosters this kind of climate. In particular, prior studies have shown that servant leadership may prevent knowledge hoarding (Song et al., 2015; Zhuang et al., 2021). Psychological safety is seen as a crucial precondition for exchanging knowledge (Edmondson et al., 2004; Zada et al., 2021), and the importance of servant leadership in enhancing psychological safety cannot be overstated (Eva et al., 2019). Through the creation of psychological safety, servant leadership is logically expected to reduce the tendency of its followers to hoard their knowledge (Sendjaya, 2015; Wu S. et al., 2021). This suggests that servant leadership is a significant antecedent to psychological safety, reducing the likelihood of knowledge hoarding. Thus, we hypothesize that:

**Hypothesis 4:** The link between servant leadership and knowledge hoarding is mediated by psychological safety.

### The Moderating Effect of Mastery Climate

According to C̀erne et al. (2014), a situational factor that effect knowledge hoarding behavior has been identified as mastery climate. Furthermore, theories of social learning and psychological safety expressly imply the presence of a mastery climate. As a consequence of the increased psychological safety given by a high mastery atmosphere, the connection between servant leadership and knowledge hoarding should be reduced. Moreover, an environment of mastery may lessen the desire to hoard knowledge (Nerstad et al., 2013). To achieve success in a mastery climate, a significant focus must be placed on teamwork (C̀erne et al., 2014). Employees actually should be less inclined to participate in knowledge hoarding as long as their actions are communicated to be publicly acknowledged, anticipated, and rewarded in this manner. A study of Poortvliet and Giebels (2012) indicates that this propensity may be ascribed to employees’ desire to continue seeking methods to develop themselves, and they are unable to admit this by hoarding information. It is possible that employees in a mastery workplace will be more motivated to recognize their self-improvement. They put greater emphasis on it, promoting their skill development by engaging in less knowledge hoarding behavior and seeking constructive cooperation. Knowledge hoarding is affected by psychological safety and mastery climate from an interactionist approach. Several factors contribute to reduced knowledge hoarding practices, such as a high level of psychological safety, an internal urge to discuss and share work-related information, and an atmosphere of mastery in the workplace (Ames and Archer, 1988). Work environments that promote, value, and reward knowledge-sharing efforts should increase the likelihood of people with high psychological safety participating in such activities (Siemsen et al., 2009). Knowledge hoarding is more likely to be practiced by people who have a poor sense of psychological safety or live in an environment that discourages the communication or sharing of information and ideas. Therefore, psychological safety encourages team members to take risks and lessens the motive for knowledge hoarding in a climate with a high level of mastery. We therefore hypothesize:

**Hypothesis 5:** A mastery climate will moderate the link between psychological safety and knowledge hoarding. The higher the level of mastery climate the weaker the relation and vice versa.

Leaders may create psychological safety in their organizations by fostering the mastery climate, attitudes, and behaviors among members of their organizations. Mastery climates—in which team members appreciate each other’s contributions, care about their well-being, and have influence into how the team works—are the most essential driver of psychological safety and therefore prevent knowledge hoarding. We hypothesized that a mastery climate would have a conditional influence on the strength of the indirect link between ethical leadership and knowledge hoarding, as seen in Figure 1, revealing a pattern of moderated mediation between the variables in our study. Specifically, we hypothesize that in a high (low) mastery climate, there is a low (high) relationship between psychological safety and knowledge hoarding.

**Hypothesis 5a:** Mastery climate will impact how servant leadership and knowledge hoarding are mediated through psychological safety; when the mastery climate is high, the indirect effect of servant leadership on knowledge hoarding will be low.

## Materials and Methods

### Sample and Procedure

We gathered data from subordinates and supervisors working in various corporate sectors in Pakistan to compile the research study data was collected from (47.23% in textile; 32.45% in information technology; 20.32% in manufacturing). The author could access the participants because of their professional and personal connections(s). One of the authors contacted the team supervisors to inform them of the study’s findings. The departments of the organizations were considered teams. The questionnaires were distributed in two parts: subordinates and the supervisors (T1 and T2). Before being delivered, the questionnaires were coded with a unique identification code to match both phases’ questionnaires. Under the condition that they could acquire a copy of the results, the teams agreed to participate. Participation was entirely optional, and respondents were guaranteed that their replies would remain anonymous. We told them that all given information will be deleted from the database to protect the participants’ privacy. The data collection was done in two rounds, each separated by 6 weeks, to minimize the possible common method biases identified by Podsakoff et al. (2003). Data collection should not be delayed for an excessively long or concise period, according to Podsakoff et al. (2012). Leadership styles and employee turnover may disturb employee’s perceptions if the time lag is too long (Babalola et al., 2017). However, if the time lag is too small, employees will go with the same approach as previous (Babalola et al., 2017). As a result, 6 weeks should be the ideal time lag to choose (Babalola et al., 2017). In phase one, 356 responses were obtained from 382 workers polled regarding servant leadership and psychological safety, knowledge hoarding and demographics (93.1%). Eighty-eight supervisors were questioned for their thoughts on the mastery climate, and we got responses out of 77 (87.5%). In the second phase, we reach out to respondents who participated in the first phase. Three hundred and fifty-six employees and 77 supervisors responded to the study and submitted their completed surveys. Respondents with missing data were excluded from the final sample (Shin et al., 2012). At last we received (287 employees and 60 supervisors) data as a final sample for analysis. Their demographic statistics show that the male participation ratio was 72.32%, with an average of 34.51 years. A total of 76.2% of employees participated with a master’s degree or above.

### Measures

#### Servant Leadership

The Servant leader scale adopted from Liden et al. (2015), was employed in the current study. It consists of 7 items with good to excellent Cronbach alpha values (α = 0.95).

#### Knowledge Hoarding

We used a 4-item scale developed by Evans et al. (2014) to assess knowledge hoarding. Sample items from this scale included “I keep news about what I am doing secret from others until the appropriate time” (α = 0.92).

#### Psychological Safety

The 5-item scale developed by Liang et al. (2012) was used to assess psychological safety. A sample item is “Nobody in my unit will pick on me even if I have different opinions” (α = 0.80).

#### Mastery Climate

We used Nerstad et al. (2013) a 6-item scale to assess mastery climate. A sample item is “In my department/workgroup, team members are encouraged to cooperate and exchange thoughts and ideas mutually” (α = 0.75).

#### Control Variables

Employee’s demographics (age, gender, and educational level) have impacted workers’ knowledge practices in the past (Connelly et al., 2012; Zhao et al., 2016; Fong et al., 2018). Thus, we controlled demographic variables in this study. Furthermore, educational levels of employees have been controlled (1 = Secondary school certificate; 2 = HSSC; 3 = master; 4 = M.Phil./Ph.D.). Employees genders were codded (Female = 0 and Male = 1).

## Results

### Descriptive Statistics

Correlation and scale reliability are shown in Table 1, together with mean values and standard deviations. All of the research variables’ correlations were in the predicted directions, as indicated in Table 1, and all of the study variables were internally consistent. The servant leadership of workers was shown to be positive correlated with psychological safety (*r* = 0.32, *p* < 0.01) and negatively related to knowledge hoarding (*r* = −0.146, *p* > 0.05). Furthermore, employees’ psychological safety was negatively related to knowledge hoarding (*r* = −0.172, *p* < 0.01). The data reliability was further tested by rho_A mechanism, the results shows (see Table 1) that all values are greater the cutoff scores of 0.7 (Dijkstra and Henseler, 2015; Henseler et al., 2015). The convergent validity was determined by evaluating factors loading, composite reliability and average variance extracted (see Table 2), all values are in acceptable range (CR < 0.7, and AVE < 0.5), thus confirming composite validity. The discriminant validity was checked through HTMT ratio, the results shows in Table 3, that all values are below than 0.85, confirming discriminant validity (Henseler et al., 2015).

**TABLE 1:** Mean, standard deviation, correlations, reliability, and rho_A.

Mean		SD	1	2	3	4	5	6	7	8	Rho_A
1. Gender	3.7291	1.003									–
2. Age	3.9107	0.928	0.042								–
3. Education	3.9539	1.113	0.115*	0.315**							–
4. Service	4.1354	1.099	0.274**	0.423**	0.413**						–
5. Servant leadership	3.7974	0.852	0.022	0.0314	0.378**	0.333**	(0.82)				(0.84)
6. Mastery climate	2.5533	1.048	0.016	0.0247	0.083	0.0175	0.308**	(0.86)			(0.87)
7. Psychological safety	4.0403	0.7396	0.043	0.0312	0.0213	0.098	0.322**	0.166**	(0.79)		(0.81)
8. Knowledge hoarding	2.4515	1.064	−0.005	0.0127	−0.025	−0.033	−0.146*	0.061**	−0.172*	(0.83)	(0.85)

*Correlation is significant at the 0.05 level (two-tailed). **Correlation is significant at the 0.01 level (two-tailed).

**TABLE 2:** Factors loadings.

Items	CR	AVE	Loadings
**Servant leadership**	**0.93**	**0.63**	
Item 1			0.81
Item 2			0.77
Item 3			0.82
Item 4			0.76
Item 5			0.81
Item 6			0.77
Item 7			0.82
Knowledge hoarding	0.85	0.60	
Item 1			0.73
Item 2			0.77
Item 3			0.82
Item 4			0.78
Item 5			0.84
Item 6			0.73
Item 7			0.79
Item 8			0.69
Item 9			0.82
Item 10			0.74
Item 11			0.86
Item 12			0.83
**Psychological safety**	0.89	0.59	
Item 1			0.76
Item 2			0.77
Item 3			0.72
Item 4			0.81
Item 5			0.73
**Mastery climate**	0.92	0.58	
Item 1			0.81
Item 2			0.77
Item 3			0.69
Item 4			0.81
Item 5			0.73

**TABLE 3:** Heterotrait–monotrait ratio of correlations (HTMTs).

Variables	1	2	3
Servant leadership	–	–	–
Knowledge hoarding	0.76	–	–
Mastery climate	0.83	0.79	–
Psychological safety	0.82	0.79	0.81

### Construct Validity

Before testing the study hypotheses, we followed (Anderson and Gerbing, 1988) recommendations and by examined the variables’ construct validity. We used AMOS 18.0 to run a series of confirmatory factor analyses (CFA) to investigate the construct uniqueness of our model’s four primary variables. Our servant leadership, psychological safety, and knowledge hoarding measurements all originated from the same source. With all fit indices falling within acceptable ranges, the four-factor model generated adequate results (χ^2^ = 213.34, CFI = 0.92, TLI = 0.91, RMSEA = 0.06, SRMR = 0.03). The four-component model was compared to a one-factor model, which comprised of a single factor (χ^2^ = 632.43, CFI = 0.57, TLI = 0.37, RMSEA = 0.42, SRMR = 0.47) (see Table 4).

**TABLE 4:** Confirmatory factor analyses and construct validity.

Model’s	RMSEA	CFI	TLI	SRMR	AIC	BIC	*X*^2^ (df)	Δ *X*^2^ (df)
Four-factor model (SL, PS, MC, and KH)	0.06	0.92	0.91	0.03	913	1254	213.34*** (81)	–
Three-factor model (SL&PS, MC, and KH)	0.21	0.62	0.73	0.22	2523	2754	511.41*** (83)	214.33***
Three-factor model (SL&MC, PS, and KH)	0.25	0.74	0.67	0.25	3256	3562	533.31*** (84)	324.21***
One-factor model (SL + KH + MC + PS)	0.42	0.57	0.37	0.47	4512	5142	632.43*** (85)	512.65***

***Correlation is significant at the 0.001 level (two-tailed). AIC, Akaike information criteria; BIC, Bayesian information criteria; CFI, comparative fit index; RMSEA, root mean square error of approximation; SRMR, standardized root mean square residual; TLI, Tucker–Lewis index.

### Common Method Variance

There is a risk of common bias while the data were gathered from a single source (Podsakoff et al., 2003). According to Chang et al. (2010), Harman’s single factor test was employed to investigate this issue. The results showed that the variation explained by a single component was 24.23%, which is far less than the 50% cutoff score. Further, to confirm the common method biasness, we compare different models with the four-factor model. The results show that our four-factor model best fits the one-factor model. This confirms that there is no issue of common method biasness in the current study (see Table 4).

### Hypothesis Testing

**FIGURE 2:**
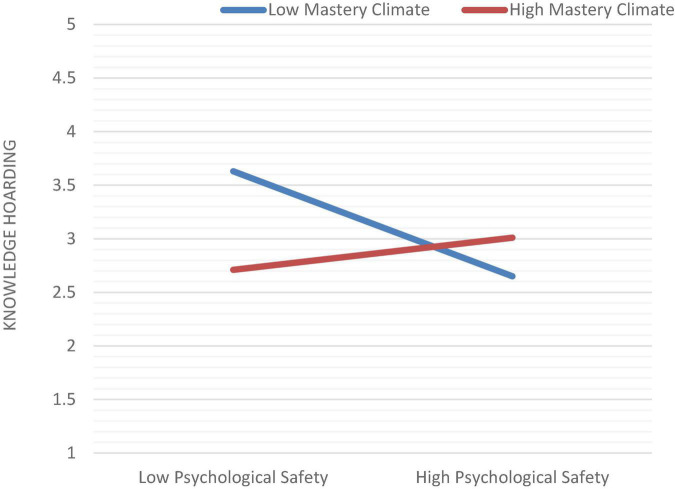
Interaction effect.

For our direct research hypothesis, we analyzed the data in Table 5. As shown in Hypothesis 1, a negative relation exists between servant leadership and knowledge hoarding (*B* = −0.057, SE = 0.67). Hypothesis 2 stated that servant leadership is positively linked with psychological safety (*B* = 0.452^∗∗∗^, SE = 0.040). Furthermore, Hypothesis 3 shows a negative association between psychological safety and knowledge hoarding (*B* = −0.104, SE = 0.077). Moreover, Hypothesis 4, which illustrates the mediation results of our study, we used the bootstrapping method (Preacher and Hayes, 2004), utilizing the Process macro Model 4 (Hayes and Rockwood, 2017). The CI for the indirect effect of servant leadership on knowledge hoarding through psychological safety does not include “0” (−0.1675, −0.0117), supporting the existence of partial mediation (see Table 6). Next, to test Hypothesis 5, we assessed the (psychological safety × mastery climate) interaction term for predicting knowledge hoarding. This interaction term is significant (β = −0.34, *p* < 0.001, CL = LLCI = −0.1675, ULCI = −0.0117) (see Table 7). The link between psychological safety and knowledge hoarding is moderated by mastery climate as a simple slop test shows in Figure 2. When mastery climate was high, the relation will be weak. To test the moderation mediation effect in Hypothesis 6, we applied (Hayes and Preacher, 2013; Hayes, 2017) macro model 7. The 95% bootstrap confidence intervals for the conditional indirect impact of servant leadership on knowledge hoarding through psychological safety at the low level (−1 SD) were generated by this approach (MacKinnon et al., 2004) and Mean level of the mastery climate did not contain zero (LLCI = 0.2157, ULCI = 0.4350), respectively, at the moderator’s high (+1 SD) level, they did not have zero (LLCI = 0.5216 ULCI = 0.8351), indicating that mastery climate serves as a moderator against the indirect effect of servant leadership on knowledge hoarding, *via* psychological safety, in support of Hypothesis 6 (Table 8).

**TABLE 5:** Regression results.

Variables	Psychological safety		Knowledge hoarding	
	*B*	SE	*B*	SE
Age	0.04	0.07	0.01	0.01
Gender	−0.06	0.05	0.02	−0.16
Age	0.01	0.02	−0.03	−0.17
Education	−0.02	0.04	0.06	0.03
Service	0.09	0.07	0.04	0.08
SL	0.452***	0.040	−0.057**	0.67
PS			−0.104**	0.077
Mediator				
Psychological safety			−0.086**	0.039
Moderator				
PS × MC			−0.349**	0.077

***Correlation is significant at the 0.001 level (two-tailed). **Correlation is significant at the 0.01 level (two-tailed).

**TABLE 6:** Mediation analysis.

Mediating variable	Effect	BootSE	BootLLCI	BootULCI
Psychological safety	−0.0860**	0.0394	−0.1675	−0.0117

**Correlation is significant at the 0.01 level (two-tailed). LLCI, lower limit 95% confidence interval; ULCI, upper limit 95% confidence interval.

**TABLE 7:** Moderation analysis.

Model	*B*	SE	*t*	LLCI	ULCI
Constant	−1.534*	0.8035	−1.9092	−3.1146	0.0463
Psychological safety	0.560**	0.1881	2.9795	0.1905	0.9305
Mastery climate	2.103***	0.3347	6.2856	1.4455	2.7622
Interaction	−0.349***	0.0771	−4.5362	−0.5011	−0.1980

*Correlation is significant at the 0.05 level (two-tailed). **Correlation is significant at the 0.01 level (two-tailed). ***Correlation is significant at the 0.001 level (two-tailed).

**TABLE 8:** Moderated mediation model.

Mediator	Level	Conditional indirect effect	SE	LLCI	ULCI
Psychological	Low	0.3253***	0.0558	0.2157	0.4350
safety	High	0.6784***	0.0797	0.5216	0.8351
	Differences	0.3531***	0.239	0.3039	0.4001

***Correlation is significant at the 0.001 level (two-tailed). Moderator values are the mean and ±1 SD, LLCI, lower limit 95% confidence interval; ULCI, upper limit 95% confidence interval.

## Discussion

Grounded on social learning theory, we examined the link between servant leadership and knowledge hoarding. The findings indicate that servant leadership and knowledge hoarding are negatively associated. Our research looked at the mediating function of psychological safety, and the findings revealed that psychological safety is a partial mediator in this relationship. A mastery climate was used as a moderator between psychological safety and knowledge hoarding, and results stated that mastery climate moderates the negative association between psychological safety and knowledge hoarding. Further, in situations where the mastery climate was strong rather than low, the indirect influence of servant leadership on knowledge hoarding *via* psychological safety was less apparent than in situations where the mastery climate was inadequate or non-existent.

### Theoretical Implications

Several theoretical additions are made to the literature on servant leadership and knowledge hoarding due to this research. First, Positive leader behaviors influence the development of knowledge hoarding practices, and our results add to a deeper understanding of this impact. Previous research on the relationship between leadership and knowledge management has mostly focused on finding successful knowledge management activities, such as knowledge sharing (Xiao et al., 2017). Servant leadership and knowledge sharing have been studied by Bavik et al. (2018). On the other hand, the effect of leadership on detrimental knowledge behaviors such as knowledge hoarding has been largely unexplored until recently (Connelly et al., 2012). Participants in their research, on the other hand, were full-time workers. This is the first research to look specifically at the relationship between servant leadership and knowledge hoarding in the workplace, and it is the first of its kind in the workplace. Second, according to the results, psychological safety was shown to be a key intervening element in the link between servant leadership and knowledge hoarding. According to social learning theory and the psychological safety viewpoint, servant leadership may contribute to the growth of employees’ psychological safety, preventing knowledge hoarding. Overall, the findings demonstrate the potential advantages of servant leadership and the fact that its impact on knowledge hoarding is mediated *via* the psychological safety of those who follow it. Third, the outcomes of this study show that the indirect link between servant leadership and knowledge hoarding through psychological safety is contingent on the existence of a mastery climate in the organization. Psychological safety has a more significant influence on knowledge hoarding in a low mastery environment than in a high mastery climate, as seen in Figure 2. Additionally, as a consequence of this study, we have been able to identify the contextual boundary elements that impact the nature of the servant leadership–knowledge hoarding relationship. This is an important addition in the body of knowledge. Fourth, this study proved that psychological safety and mastery environment affect knowledge hoarding. It also looked into mastery climate as a mediator in the link between psychological safety and knowledge hoarding. Lastly, with Edwards and Lambert (2007) moderated mediation technique, we observed that psychological safety has a considerable impact on the relationship between servant leadership and knowledge hoarding, depending on the level of mastery climate in the organization.

### Practical Implications

The findings of our research also have managerial implications. First, we urge managers to put the needs of their teams and organizations ahead of their interests. Managers do not place a high value on achieving their personal goals. They need to help their employees to attain a goal (Khan et al., 2022c). Organizational progress and well-being should be manager’s primary concern, not personal gain. On the other hand, traditional leadership is characterized by the amassing and exercise of authority by a person at the “head of the pyramid.” The servant-leader shares authority prioritizes the needs of others, and encourages employees to reach their full potential. This kind of endeavor is beneficial since it can increase the psychological safety of each employee. Workers who report feeling secure in their positions are less likely to engage in knowledge hoarding. Organizations can provide training programs to cultivate leaders’ professionalism give examples of serving conduct that leaders should demonstrate in their management policies and day-to-day behavior. Establish formal and informal mentoring programs to assist leaders in improving their serving leadership abilities. Second, the findings of our study indicate that a mastery atmosphere seems to be an ideal work environment for lowering employees’ propensity to hoard their knowledge. Organizations may reduce the incidence of knowledge hoarding practices by cultivating a mastery environment that encourages learning, cooperation, and skill development. Managers, for example, may foster a mastery atmosphere by offering particular training and development programs that enable workers to acquire job-related abilities, recognize the importance of teamwork, and recognize the conditions for success and failure during task completion. Also, to facilitate communication and knowledge exchange, managers might establish institutionalized platforms or channels. They may be advantageous in developing a mastery climate, which will prevent the occurrence of knowledge hoarding from happening. Third, the outcomes of our research demonstrated that psychological safety plays an essential role in controlling the relationship between servant leadership and knowledge hoarding in organizations. To protect the psychological safety of their employees, managers should take proactive measures. Psychological safety is dynamic and may be enhanced *via* healthy leader-member interactions (Frazier et al., 2017). Managers, for example, should communicate with workers openly and transparently and offer them a psychologically stable workplace. The perceived psychological safety of workers will increase due to this, and knowledge hoarding practices will be reduced.

### Limitations and Suggestions for Future Research

Every study has some limitations that should be addressed in the future. This study also has some limitations. First, this study was examined at individual and team levels. Therefore, we have to control demographics at both levels. However, organizational culture plays a crucial role in knowledge management (i.e., knowledge sharing, knowledge hoarding) and effect employees knowledge hoarding behavior. Future research should control organization culture. Second, our sample is from Pakistani corporate culture, where trust matters among individuals compared to other organizational factors. Many organizations are family-run and have reference base jobs in some scenarios. Our study results are therefore not generally applicable to other countries. The different organization have a different culture that influences employees. Accordingly, we suggest that the same conceptual model be tested in other settings. We have grounded this study based on social learning theory and examined the link between servant leadership and knowledge hoarding. Further, it is suggested to link other approaches to this model, i.e., social cognitive theory. It is also suggested that other potential mediators should be used in the future, i.e., psychological capital and psychological empowerment may minimize employees’ intention toward knowledge hoarding. In addition, we have used mastery climate as a potential moderator in this study because such climate believes in support, cooperation and emphasis on team and individual development. Further, it is suggested to study other potential moderators, i.e., organization commitment and interpersonal trust. For example, Connelly et al. (2012) indicated that when employees are committed to organization are less likely to hoard knowledge, because they view responding to coworkers’ requests as their professional responsibility. To effectively create, share, and utilize knowledge in teams, individuals must trust one another. To be successful in a team environment, it is critical to have complete faith in the group’s ability to work together and share information. These processes are influenced by the degree of interpersonal trust relationships.

## Conclusion

Effective knowledge management is impossible without effective leadership. A leader is thus the one who should establish an organization that fosters the development, sharing, and application of new knowledge inside organizations. This study provides a negative association between servant leadership and knowledge hoarding. Servant leadership play a key role in knowledge sharing among employees. Further, psychological safety mediates this relationship significantly. Furthermore, this study illustrates that mastery climate plays a moderating role in between psychological safety and knowledge hoarding, presence of mastery climate weakens the link between psychological safety and knowledge hoarding. The integrated model illustrates that the importance of servant leadership that encourage and cultivate safe atmosphere to prevent knowledge hoarding in the organization. This study is important to body of knowledge by introducing new leadership style with knowledge hoarding, that is unexplored till date. With these findings in mind, this work serves as a helpful study for further research into additional components and processes that impede knowledge hoarding.

## Data Availability Statement

The raw data supporting the conclusions of this article will be made available by the authors, without undue reservation.

## Author Contributions

SZ, JK, ZJ, and IS contributed to the conception and design of the study. SZ organized the database. JK performed the statistical analysis. SZ, JK, and IS wrote the first draft of the manuscript. ZJ, AV-M, and NC-B wrote sections of the manuscript. All authors contributed to manuscript revision, read, and approved the submitted version.

## Conflict of Interest

The authors declare that the research was conducted in the absence of any commercial or financial relationships that could be construed as a potential conflict of interest.

## Publisher’s Note

All claims expressed in this article are solely those of the authors and do not necessarily represent those of their affiliated organizations, or those of the publisher, the editors and the reviewers. Any product that may be evaluated in this article, or claim that may be made by its manufacturer, is not guaranteed or endorsed by the publisher.
